# Biological differences underlying sex and gender disparities in bladder cancer: current synopsis and future directions

**DOI:** 10.1038/s41389-023-00489-9

**Published:** 2023-09-04

**Authors:** Bhavisha Doshi, Sarah R. Athans, Anna Woloszynska

**Affiliations:** Department of Pharmacology and Therapeutics, Roswell Park Comprehensive Cancer Center, Buffalo, NY 14203 USA

**Keywords:** Bladder cancer, Cancer genetics

## Abstract

Sex and gender disparities in bladder cancer have long been a subject of interest to the cancer research community, wherein men have a 4 times higher incidence rate than women, and female patients often present with higher-grade disease and experience worse outcomes. Despite the known differences in disease incidence and clinical outcomes between male and female bladder cancer patients, clinical management remains the same. In this review, we critically analyze studies that report on the biological differences between men and women and evaluate how these differences contribute to sex and gender disparities in bladder cancer. Distinct characteristics of the male and female immune systems, differences in circulating hormone levels and hormone receptor expression, and different genetic and epigenetic alterations are major biological factors that all likely contribute to disparate incidence rates and outcomes for male and female bladder cancer patients. Future preclinical and clinical studies in this area should employ experimental approaches that account for and consider sex and gender disparities in bladder cancer, thereby facilitating the development of precision medicine for the effective treatment of bladder cancer in all patients.

## Introduction

Sex and gender disparities exist in all facets of healthcare and disease and can arise as a result of several factors, including social, behavioral, and biological determinants of health [1]. Biological differences that drive health disparities can lead to altered clinical outcomes for male and female patients. Therefore, it is crucial to have a thorough understanding of the biological differences between men and women in order to develop more personalized preventative measures and treatment strategies for different diseases. Here, we focus on sex and gender disparities in bladder cancer and define these as any difference in cancer measures between male and female patients, such as variation in incidence, prevalence, morbidity, mortality, and stage at diagnosis. Gender is a social construct defined by an individual as a sense of being male, female, neither, both, or another gender [2]. Sex, biological sex, or sex assigned at birth, is an assignment or classification of a person as male, female, intersex, or another sex based on anatomy, hormone levels, and chromosomes [2]. We recognize that there are several genders that exist outside of the male/female dichotomy, but limited studies provide information outside of self-reported men or women. Therefore, for the purposes of this article, we will investigate disparities between people identifying as women/female compared to people identifying as men/male, in addition to differences between biological sexes as discerned by gonadal anatomy, hormone levels, or sex chromosomes. Global cancer analyses have shown that male-gender are four times more likely to develop bladder cancer than female gender; however, female-gender patients diagnosed with bladder cancer have a higher risk of recurrence, progression, and cancer-specific mortality compared with their male counterparts [3]. Bladder cancer is the 10th most common cancer worldwide, with an estimated 75,000 new cases and 16,700 deaths each year in the United States [4]. Carcinogen exposure from tobacco consumption accounts for the cause of approximately 50–65% of bladder cancer cases in men and 20–30% of bladder cancer cases in women [5]. It is long believed that the high incidence of bladder cancer in male-gender is due to high smoking rates in men compared to female-gender [6]. However, the incidence of bladder cancer remains high in male-gender compared to female-gender even after adjusting for smoking status [5]. Less than 10% of bladder cancers are attributed to occupational carcinogen exposure [7], and therefore differences in occupation between men and women do not explain the significant gender disparities that exist.

Bladder cancer also referred to as urothelial cancer, can be separated into two categories depending on the depth of invasion into the bladder wall. Noninvasive bladder cancer includes carcinoma in situ and non-invasive papillary carcinoma, and non-muscle invasive bladder cancer (NMIBC) is confined to the lamina propria. Conversely, muscle-invasive bladder cancer (MIBC) invades through the muscle and beyond into the tissue surrounding the bladder. Bladder tumors can be further characterized as either luminal or basal based on molecular subtyping. Luminal bladder tumors tend to have papillary features and express markers associated with urothelial differentiation, whereas basal tumors express markers of the basal layer of the urothelium and show more frequent squamous differentiation [8].

**Fig. 1:**
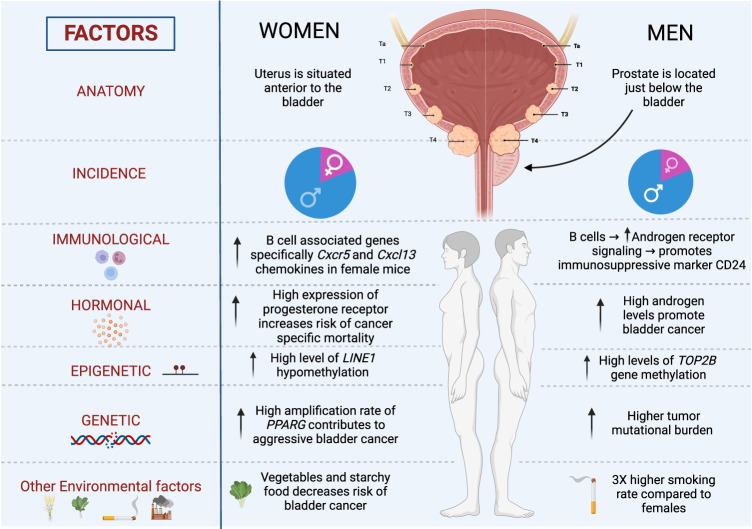
Summary of factors associated with bladder cancer sex and gender disparities. Differences between women and men, which may play a role in bladder cancer, including differences in anatomical features, incidence rate of bladder cancer, the immune system, hormonal expression, the epigenome, are depicted on the left and right sections, respectively. Created with BioRender.com.

The present review aims to critically analyze studies that report on differences between men and women in both the healthy and tumor-bearing states and analyze how these different characteristics (summarized in Fig. 1) may contribute to sex and gender disparities observed in bladder cancer. A comprehensive understanding of these differences has the potential to guide the development of clinical strategies to eliminate these health disparities where possible.

## Risk factors that act in concert with sex and gender to influence bladder cancer incidence and mortality

### Age

Age is the top independent risk factor for bladder cancer, and the median age at diagnosis is approximately 70 years [9]. Patients diagnosed at an older age are more likely to have high-grade malignancies, whereas younger patients are more likely to have lower-grade, lower-stage, non-muscle invasive disease [10, 11]. Furthermore, it has been clearly demonstrated that older age predicts worse survival and a higher risk of recurrence for both NMIBC and MIBC patients [12, 13]. Older patients are less likely to receive neoadjuvant chemotherapy for MIBC because of contraindicatory factors like the patient’s fitness and life expectancy, which may contribute to altered clinical outcomes [14]. Female-gender patients tend to present with bladder cancer at an older age compared with male-gender patients, which may contribute to the higher frequency of high-stage and grade disease in these patients [15]. Older age is associated with an overall increase in the frequency of metastatic dissemination for both male and female-gendered patients. Specifically, rates of bone, brain, lung, and liver metastases increase with age, while lymph node metastasis is more prevalent in younger patients [16].

Bladder cancer patients with liver metastasis have worse survival outcomes when compared to patients with bone, brain, and lung metastasis [17]. In male-gender patients, the increase in bone and brain metastases with age is significantly higher, whereas the increase in lung and liver metastases with age is significantly higher in female-gender patients [16]. Increased frequency of liver metastasis in female-gender patients older than 80 years old compared to female-gender patients under 63 years old may partially explain the worse survival outcomes in female-gender bladder cancer patients.

The mechanisms underlying gender and sex-specific metastatic sites of bladder cancer remain unknown. Interestingly, phosphoinositide 3-kinase (PI3K) signaling in bladder cancer promotes bone metastasis by regulating the transcription of Zinc finger E-box-binding homeobox 1 (ZEB1) [18]. Additionally, androgen stimulation of bladder cells increases the activity of the PI3 kinase/AKT signaling pathway [19]. Generally, the male sex has higher levels of circulating androgens compared to the female sex due to gonadal differences [20], which may thus promote PI3K signaling and contribute to the increased rates of bone metastasis in male bladder cancer patients.

### Race and ethnicity

Race and ethnicity significantly impact bladder cancer risk and clinical outcomes, as indicated by differences among European American (EA), African American (AA), Hispanic, and Asian and Pacific Islander (API) patients. EA patients comprise 75.5% of new bladder cancer diagnoses [21]. However, AA patients tend to present with more advanced stage, higher grade, muscle-invasive, or metastatic disease [21, 22]. This may be partially explained by the fact that AA patients, regardless of socioeconomic status and availability of medical care, are less likely to receive urology referrals, cystoscopy, or imaging and are diagnosed at an older age [23]. Also, AA MIBC patients are less likely to receive the standard treatment regimen, i.e., neo-adjuvant chemotherapy plus cystectomy [21]. Differences in disease presentation, diagnosis, and treatment all contribute to an increased risk of mortality and shorter survival for AA bladder cancer patients [24–26]. There are many race-specific differences in tumor biology that likely contribute to racial disparities in bladder cancer. For example, tumor localization within the bladder differs significantly between EA, AA, and API patients [21]. While EA tumors localize to the lateral wall, trigone, and ureteric orifice of the bladder, AA tumors localize to the dome and anterior wall of the bladder, and API tumors are most frequently on the posterior wall of the bladder, the bladder neck, and the urachus [21]. Also, AA patients present with the largest average tumor size when compared to API patients, and the rate of tumor-positive lymph nodes is higher in AA patients than in EA patients [21]. EA patients have higher mutation rates in genes known to promote bladder cancer initiation and progression, including *TP53*, *ARID1A*, *ERBB3*, and *CDKN1A*, compared with AA patients [27]. A higher frequency of these mutations may predispose EA patients to bladder cancer, potentially explaining why the incidence rate is so high in the EA population specifically. In relation to sex and gender, AA female-gendered patients have the highest rates of muscle-invasive and metastatic disease, demonstrating that gender plays an important role in bladder cancer disparities in addition to race [25, 28, 29]. It is likely that race-specific and gender-specific differences in bladder tumor biology cooperate to promote the disparities that are observed in bladder cancer.

### Diet

A person’s diet influences the relative risk of developing bladder cancer [30]. For example, a diet rich in fruits, vegetables, and fermented dairy products is associated with a reduced risk of bladder cancer [31–33]. Interestingly, evidence suggests that dietary intake of certain foods can differentially affect the risk of bladder cancer in men compared with women. A study by Yu et al. reported that a higher intake of vegetables and non-starchy foods like broccoli, garlic, cauliflower, kale, cabbage, and asparagus reduced bladder cancer risk in female-gender but had no impact on the risk in male-gender [34]. Additionally, the intake of at least one avocado per week is associated with a decreased risk of bladder cancer in male-gender but has no impact on the risk of developing bladder cancer in female-gender [35]. Another study, published around the same time, found that a higher intake of sugary drinks increased the risk of bladder cancer in female-gender but had no impact on the risk in the male-gender [36]. The exact mechanisms behind the differential effects of consuming certain foods or beverages on bladder cancer risk between sexes and genders remain unknown. However, one possible explanation may relate to differences in fuel metabolism between men and women. Women derive more energy from fat oxidation, whereas men derive more energy from carbohydrate oxidation [37]. Female-gender have high urine concentrations of glycine [38]. Vasudevan et al. demonstrated injecting glycine in rats induces bladder tumorigenesis [39], which suggests that elevated levels of glycine might induce tumorigenesis. Female-gender also has higher concentrations of some non-canonical amino acids like acetyl phenylalanine, 2-aminoadipic acid, N-acetyl aspartic acid, nicotinuric acid, aminosalicyluric acid in their urine [40], whereas male-gender have high concentrations of proline [40], leucine [41], l-carnosine, and 2,6-diaminopimelic acid [42]. Carnosine possesses antitumor activity against bladder cancer both in vitro and in vivo through the regulation of tumor angiogenesis [43]. Male-gender has high urine carnosine levels compared to female-gender [42]. High carnosine concentrations in the urine of male-gender thereby may slow bladder cancer from progressing to higher stage disease compared to female-gendered bladder cancer patients. Conversely, high levels of leucine may promote the initiation of bladder cancer in the male sex, as it promotes bladder tumor initiation in rats [44].

## Clinical aspects of sex and gender differences in bladder cancer

### Tumor characteristics

Tumor characteristics include tumor stage, grade, metastasis, and histology. The tumor stage is determined based on the TNM classification system, where T indicates the size of the primary tumor and invasion into the bladder wall, N is the extent of regional lymph node involvement, and M is the presence of distant metastasis [45]. Compared with age-matched male-gender patients, female-gender patients present with higher-stage bladder cancer (T2 and higher) on primary diagnosis and are more likely to have N+ and M+ staged disease [25, 26, 46]. Female-gender patients also have higher-grade disease compared with male-gender patients on initial diagnosis [28]. Several studies have also shown that female-gender bladder cancer patients are more likely to experience recurrence or progression compared with male-gender patients with matched stage and grade disease [47, 48]. These findings suggest that female-gender bladder cancer patients tend to have worse disease presentation at the time of diagnosis and have poor outcomes compared to their male counterparts. Thus, these findings emphasize the importance of biological differences between men and women in bladder cancer, which are manifested in distinctly disparate tumor characteristics. Understanding the causative factors for the observed disparities between male and female tumors would thereby help in determining an optimized treatment plan for better outcomes and improved survival.

### Consequences of sex and gender-specific differences during the diagnosis and treatment of bladder cancer

The first symptom of bladder cancer that prompts evaluation is hematuria [49]. Female-gendered patients are two times less likely than male-gendered patients to see a urologist prior to any incidence of hematuria [23]. Even after the presence of hematuria, female-gendered patients are less likely than male-gendered patients to be referred to a urologist and less likely to have a cystoscopy, which may contribute to delays in the evaluation and diagnosis of bladder cancer [23, 50]. These differences remain significant after adjusting for demographic factors, availability of medical care, and bladder cancer risk factors. This suggests the possibility that societal gender bias may be a cause of differences in the evaluation and diagnosis.

Bladder cancer treatment varies with tumor stage and risk stratification. The first course of treatment for NMIBC patients (tumor stage Ta, Tis, or T1) is surgical trans-urethral resection of the bladder tumor (TURBT). Low- and intermediate-risk patients, encompassing most low-grade and high-grade stage Ta tumors as well as low-grade T1 tumors, are given chemotherapy to clear remaining tumor cells within the bladder after TURBT. The recommended treatment for high-risk NMIBC patients, which includes high-grade T1 and high-grade recurrent or large Ta tumors, is intravesical bacillus Calmette–Guérin (BCG) treatment. For MIBC patients (stage T2 and higher), the current standard-of-care treatment includes neoadjuvant systemic chemotherapy, which can include methotrexate, vinblastine, doxorubicin, cisplatin, gemcitabine, or a combination of chemotherapies, followed by radical cystectomy.

Meta-analysis of 31 studies by Mori et al. revealed that there is no gender-specific difference in the overall survival, recurrence-free survival, and progression-free survival in NMIBC patients after surgical removal of tumor and BCG treatment [51, 52]. However, the meta-analysis of 63 studies of MIBC patients showed a gender-specific difference in overall survival, recurrence-free survival, and progression-free survival [51]. These gender-specific differences in MIBC might be due to the treatment, as few studies have shown that female-gendered patients are less likely to obtain systemic chemotherapy [53]. However, female-gendered patients are more likely to receive radical cystectomy, a surgical procedure that includes the removal of the whole urinary bladder and nearby lymph nodes [15]. After adjusting for receipt of either treatment, survival outcomes for female bladder cancer patients remain lower than their male counterparts [15, 53]. More recent data indicate that female-gendered and male-gendered MIBC patients are equivalently likely to complete neoadjuvant chemotherapy prior to cystectomy, which may be due to current efforts toward the attainment of the highest level of healthcare for all people, regardless of gender, race, ethnicity, etc. [54]. Although receiving equal treatment regimens as male-gendered patients, female-gendered bladder cancer patients continue to experience poor survival outcomes. This suggests that differences in tumor biology may be the major driver of these disparate outcomes rather than differences in treatment.

## Biological differences underlying sex and gender disparities in bladder cancer

### Immune system differences between healthy males and females

The immune system acts as the human body’s protector from infection and disease. In a healthy state, many facets of the immune system differ between male-sex and female-sex humans [55, 56]. For example, there are significant differences in the distribution and abundance of immune cell populations in the body between sexes. The female sex has a lower percentage of natural killer (NK) cells but a higher percentage of plasma cells in peripheral blood, indicating that the female sex may have a stronger antibody immune response [55]. Specifically, female-sex immune cells express higher levels of interferon-gamma (IFNɣ), lymphotoxin beta (LTB), and granzyme A (GZMA), which are inflammatory genes that allow immune cells to neutralize pathogens [55]. This suggests that T cells derived from the female sex are activated to a higher extent than those derived from the male sex. Many of the differentially expressed immune response genes have estrogen receptor (ER) response elements in their promoters [55], which may imply that higher estrogen levels in humans with female gonads are driving differences in the expression of the immune response. Early studies of human lymphoid and murine splenic cells revealed that treatment with estradiol can lead to an increase in *IFNɣ* promoter activity in ER-positive cells, with a concomitant increase in *IFNɣ* mRNA expression [56]. Thus, higher estrogen levels intrinsic to people with female gonads may lead to upregulated immune responses via estrogen-mediated expression of *IFNɣ*. In a non-cancer setting, this increase in cytotoxic effector genes can have an adverse effect on health and lead to an increased incidence of autoimmune disease in people with female reproductive organs [55]. The exact mechanism that protects the female sex from developing bladder cancer is yet unknown, however, the enhanced immune response in the female sex might be beneficial in the context of cancer, as it may provide anti-tumor immunity.

Androgens, like testosterone, play a role in immune regulation by impacting various aspects of the immune system, including T cell proliferation, NK cytotoxicity, decreasing antibody production, and stimulating the production of anti-inflammatory cytokines [57]. The influence of androgens on the immune system is greatly reviewed by Ben-Batalla et al. [57], and therefore our review focuses on the interplay of androgens and the immune system that may lead to sex/gender disparity in bladder cancer. Specifically, the addition of testosterone in vitro in blood cells stimulates T cells to produce Th1 cytokines and a high Th1:Th2 cytokines ratio in male cells [58, 59]. High Th1 cytokine is protective against bladder cancer [60]. Since people with male reproductive organs have high testosterone levels, this may cause an increase in the production of Th1, and thus these patients may develop relatively less adverse diseases compared to their female counterparts. The crosstalk between progesterone and the immune system has not yet been investigated in bladder cancer. In general, progesterone is known to be immunosuppressive [61].

Studies in rodents have shown that the male sex has fewer tissue-resident immune cells than the female sex, including T cells, B cells, and macrophages [62]. Specifically, CD4^+^ and CD8^+^ tissue-resident T cells were significantly higher in female rodents compared with male rodents in both mice and rats, whereas there was no difference in CD4^+^CD25^+^ regulatory T cells [62]. Female-sexed tissues from rodents express higher levels of chemokines, such as Cx3xl1, Ccl2, Cxcl12, and Ccl5, which attract immune cells. Female-sexed leukocytes also have increased expression of chemokine receptors Ccr1, Ccr2, and Cxr4 [62]. This highlights the differential expression of both chemokines and their receptors in female-sexed animal models, which ultimately leads to larger populations of tissue-resident immune cells. These data from animal models suggest that tissue-resident immune cells may be another possible mechanism protecting female sex from developing cancer. This also supports the fact that the male sex has a higher probability of developing cancer compared to the female sex [63].

Viral immunity and cytokine responses differ between male-sex and female-sex as well [64, 65]. In a study of cytomegalovirus-positive cells, which is common in humans, several cytokines were differentially detected between male and female cells after in vitro immune stimulation. Overall, expression levels of IL-2 and IFNɣ were significantly higher in female-sex-derived immune cells, whereas male-sex-derived immune cells expressed significantly higher amounts of TNF-ɑ, an inflammatory cytokine that induces apoptosis [64]. This emphasizes the importance of studying both overall and individual immune cell populations, as gender and sex-specific differences exist.

### Differences in the immune system that influence tumor immunology

Avoidance of immune destruction is one of the hallmarks of cancer [66]. The intrinsic differences in the immune system and immune response between men and women likely contribute to gender disparities in cancer. Although sex and gender-based differences in tumor immune responses are not well studied in bladder cancer, they have been well-documented in other cancer types [67, 68].

Liver cancer, for example, is 3–5 times more common in male-gender compared with female-gender [69]. Levels of IL-6, which promotes tumor growth, are increased in liver cancer patients. One of the mechanisms that protect biologically female liver cancer patients is estrogen-mediated blockage of IL-6 by the Kupffer cells of the liver [67]. Male-gender patients tend to have more proliferative tumors caused by elevated production of cortisol, an immune suppressive steroid hormone, increased infiltration of tumor-associated macrophages (TAMs), and increased infiltration of tumor-associated neutrophils (TANs), both of which are pro-tumorigenic [45].

In non-small cell lung carcinoma (NSCLC), female-gender patients produce a stronger anti-tumor immune response than male patients, and they have a higher number of activated dendritic cells, CD4^+^, CD8^+^, and effector T cells, memory CD4^+^ T cells, and B cells [68]. When treated with anti-PD1/PD-L1 immunotherapy plus chemotherapy, female-gender NSCLC patients survive longer than their male-gender counterparts [68]. PD-1 and its ligand PD-L1 are immune-repressive markers expressed in T cells and tumor cells, respectively, which stop the ability of T cells to bind and recognize foreign antigens protecting the tumor from detection [70, 71].

The interplay between hormones and the immune system may account, at least in part, for gender disparities in cancer. For example, androgen receptors (ARs) promote the expression of CD24, an immunosuppressive surface marker on tumor cells [72]. Additionally, bladder cancer cells have been shown to recruit more B cells than normal bladder cells. These tumor-recruited B cells promote AR signaling pathways, which further promote the expression of genes associated with metastasis, such as *MMP1* and *MMP13* [73]. As people with male reproductive organs have higher levels of circulating androgens compared to those with female reproductive organs, these data suggest that the male sex may have a more immunosuppressive and tumor-promoting tumor microenvironment (TME).

Several mouse models recapitulate gender disparities in human disease [74–77], thereby allowing for the study of sex-based differences and their impact on the immune system. In an epithelial squamous cell carcinoma mouse model, female mice, upon exposure to a carcinogen, activate immune response pathways and upregulate CD4^+^ and CD8^+^ T regardless of any mutation in DNA caused by the carcinogen [78]. However, the male-sexed mice, upon exposure to a carcinogen, show a higher rate of mitosis and proliferation of cancer cells, which leads to less differentiated, higher-stage epithelial squamous cell carcinomas [78]. These findings indicate that immune responses may endow female-sexed mice with some degree of protection against carcinogen exposures which is highly relevant to bladder cancer. Furthermore, the studies in the in vivo models suggest that there are differences in the tumor’s immune response between males and females [74–79]. Thus, the immune response may represent a major biological factor that contributes to sex and gender disparities in cancer patients. The differences in tumor immune biology of male and female bladder cancer patients thereby become an important facet to study. Existing immunocompetent mouse models, like the N-butyl-N-(4-hydroxy butyl)-nitrosamine (BBN) spontaneous bladder tumor model [80], will be useful to specifically study the differences between the male-sex and female-sex bladder TME. Studies in the BBN bladder cancer model indicated that female-sexed mice have an increased expression of B cells associated genes, including *Cxcr5*, *Cxcl13*, *Cd19*, *Cd79a*, *Pax5*, *Mzb1*, *Ms4a1*, and tertiary lymphoid structures [81]. This suggests that females produce a more robust anti-tumor immune response in the context of bladder cancer.

### Androgen and estrogen as drivers of bladder cancer

Hormones alter the TME, immune response, and tumor cell biology as a result of their interactions with the appropriate receptor and induction of respective signaling pathways. Several hormones, like androgen, estrogen, and progesterone, affect bladder cancer initiation and progression [82]. The sex hormones—androgen, estrogen, and progesterone circulate in different levels between people with male and female reproductive systems.

AR expression in bladder cancer cells is associated with the chemoresistance observed in bladder tumors [83]. In AR-positive bladder cancer cells, excess androgen decreases cell sensitivity to cisplatin, whereas AR-negative bladder cancer cells are significantly more sensitive to cisplatin [83]. Cisplatin-based chemotherapy is frequently used as a first-line treatment for MIBC patients. Therefore, tumor cells expressing AR or in an environment rich with androgens may be less responsive to cisplatin-based treatment. In carcinogen-induced bladder cancer models, castration or anti-androgen treatment significantly decreased the initiation of bladder cancer [84]. Further, treatment with androgen deprivation therapy, anti-androgens, or small interfering RNA (siRNA) against AR decreased proliferation and tumor growth in animal models of bladder cancer [72, 73]. These studies provide limited data because only male-sexed mice were used. Future studies should include both male-sexed and female-sexed animals to identify whether the effects of decreased androgen levels and decreased AR activity are sex-specific. An in vivo study utilizing the BBN mouse model, a carcinogen-induced model of bladder cancer, revealed a possible mechanism by which AR contributes to sex differences in bladder cancer. The study showed that AR directly regulates the expression of *Tcf7*, thereby leading to the exhaustion of CD8^+^ T cells. Tcf7 is a transcription factor involved in the early fate decision for the activation of CD8^+^ T cells. Exhaustion of CD8^+^ T cells impairs tumor immunity and contributes to the uncontrolled growth of the tumor [85]. Therefore, it is possible that both androgen levels and AR expression may have a tumor-promoting effect in bladder cancer, and both potentially contribute to the observed disparities in incidence between male-sex and female-sex.

The impact of estrogen levels on bladder cancer, however, remains unclear. Female-gender patients who have used estrogen therapy have a significantly lower risk of developing bladder cancer [86]. This suggests that an increased level of female sex hormones may have a protective effect against bladder cancer. However, bladder cancer patients with female reproductive organs who have had children and who reported a late age (>15 years) at menarche, both of which decrease lifetime estrogen production, also have a decreased risk of developing bladder cancer [87, 88]. The estrogen ligand has two main nuclear receptors, namely, ER alpha (Erα) and ER beta (Erβ). Erβ is more prevalent in bladder tumors than Erα, and Erβ expression is significantly higher in high-grade and muscle-invasive bladder cancer [89]. In vitro, treatment with anti-estrogens or knockdown of ERβ decreases the proliferation and invasion of bladder cancer cells, while overexpression of ERβ has the opposite effect [90, 91]. Although both ERβ and AR seem to influence bladder cancer, they are expressed at similar levels in the bladder tumors of patients with male and female gonads [92]. Interestingly, the progesterone receptor (PR) is expressed at higher levels in female-gender bladder tumors [93], and PR positivity is associated with a higher risk of cancer-specific mortality [94]. In general, progesterone is known to be immunosuppressive [61]. Thus, high expression of progesterone and PR in female-gender bladder tumors can be one of the reasons for the observed higher mortality rates for female patients.

Overall, the mechanistic role of androgen, estrogen, and progesterone in bladder cancer remains unclear. The difference in the disease progression and outcome of male and female-gendered bladder cancer patients can be partially explained by the crosstalk between hormones and other biological factors. For example, the ER has been shown to regulate TNF-ɑ expression in dendritic cells and repress TNF-ɑ expression in cancer cells [95]. As TNF alpha is tumor-promoting [96, 97], the estrogen-mediated repression of TNF alpha may support a stronger anti-tumor immune microenvironment in bladder tumors of patients with female gonads.

### Differences in genetics and metabolism

#### Effects of sex chromosomes

Chromosomal sex is defined by the presence of X and Y chromosomes. The male genome contains one X and one Y chromosome, and loss of the Y chromosome is frequent in bladder cancer [98]. Y chromosome loss is associated with an increased risk of cancer-related mortality in general [99]. While Y chromosome loss is not associated with changes in tumor growth, tumor grade, and tumor stage of bladder tumors, specifically [100], recent work shows that deletion of the Y chromosome by CRISPR–Cas9 contributes to immune dysfunction in the TME and better response to immune checkpoint blockade [101]. Therefore, it is not clear what role the Y chromosome plays in preventing or protecting against bladder cancer. The female genome contains two X chromosomes, and one X chromosome is silenced by Xist [102]. In both normal and tumor cells, certain genes, such as *KDM6A* [103], escape the X-chromosome inactivation process and become expressed at significantly higher levels in individuals of female chromosomal sex compared to male chromosomal sex [104]. KDM6A is known to have tumor-suppressive activity [103]. *KDM6A* is highly expressed in women when compared to men, yet the mutation frequency of KDM6A is higher in female-gendered bladder cancer patients [103, 105]. In normal bladders, it is possible that high *KDM6A* expression due to female chromosomal sex may protect women from developing bladder cancer as frequently as men. Then, if *KDM6A* acquires a mutation before or after tumor development, it could possibly drive higher-grade tumors in patients with female chromosomal sex. Future studies should investigate the consequences of overexpression due to X-inactivation escape to determine unique vulnerabilities in bladder cancer cells.

#### Differential mutations and gene expression

Compared with other cancers, bladder tumors exhibit a high number of genetic mutations [106]. These mutations can upregulate, downregulate, or completely eliminate the expression of the gene in which they occur or lead to the translation of proteins with altered structure and function. The genes most commonly amplified in bladder cancer include *AHR, BCL2L1, CCND1, CCNE1, E2F3, EGFR, ERBB2, FGFR3, GATA3, KRAS, MDM2, MYCL1, PPARG, PVRL4, SOX4, TERT, YWHAZ*, and *ZNF703*, whereas the most commonly deleted genes are *TP53, FGFR3, KDM6A, STAG2, CDKN2A*, and *RAD51B* [106]. The frequency of specific types of DNA mutations differs between male-sex and female-sex bladder cancer patients. Specifically, male-sex bladder cancer patients have more single nucleotide variants and a higher tumor mutational burden when compared with their female-sex counterparts [107–109]. Differences in the incidence of mutations between male-sex and female-sex bladder tumors can be influenced by extrinsic factors. For example, carcinogens ingested during cigarette smoking are associated with specific mutational signatures across almost all human cancer types, including bladder cancer [110]. Furthermore, genes that are exclusively expressed in biological males, such as the testes exclusive genes *GPR64* and *SPINT3*, accumulate deleterious mutations [111]. Depending on the affected gene, these mutations may predispose biological males to cancer. In the case of bladder cancer, this could partially explain the higher incidence rate in the biological males.

There are several genes that are differentially mutated or differentially expressed in a gender-specific manner in bladder tumors. *PPARG*, a nuclear receptor that regulates lipid and glucose homeostasis, cellular energy use, and mitochondrial function [112], is one of the most amplified genes in muscle-invasive bladder cancer. Tumors from female-gender bladder cancer patients have high mRNA expression of *PPARG* [113] and a higher level of PPARG receptor expression when compared with their male-gender counterparts. Higher levels of PPARG are associated with late-stage and more aggressive bladder cancer [112, 114]. *TP53* is a tumor suppressor gene that is commonly mutated in almost all human cancers [115]. In bladder cancer patients, male-gender patients have a higher mutation frequency in the *TP53* gene when compared with female-gender patients [116]. The *TP53* mutations are mostly loss-of-function mutations and are classified as driver mutations in bladder cancer [115]. *TP53* mutations in bladder cancer are associated with cancer initiation, increased metastasis, disease progression, and proliferation [115, 117]. *KDM6A* is one of the most frequently mutated genes in bladder cancer [103, 105]. *KDM6A* is located on the X chromosome and is known to escape the X-chromosome inactivation process, thereby providing females with two functional copies of the gene [118]. In bladder cancer, *KDM6A* mutations lead to lower numbers of tumor-infiltrating immune cells, increases in inflammatory signaling, and promotion of tumor immune escape [119–122], which facilitates tumor progression. *KDM6A* has been found to be more highly mutated in female-sex bladder cancer patients than in male bladder cancer patients [103, 105].

Overall, differences in genetic mutations found in male and female bladder tumors are likely important contributors to the variation in tumor characteristics and patient outcomes. Increased mutational frequency of *KDM6A*, in addition to high expression of PPARG, may drive more aggressive, higher-stage bladder tumors in female-gender patients, whereas frequent mutations in *TP53* may contribute to increased incidence of bladder cancer in male-gender patients. Additional investigation into gender and sex-specific mutations and gene expressions between male and female bladder tumors would be worthwhile, especially with current genomics and proteomics technologies. These efforts may uncover potential proteins or pathways that can be targeted specifically in male or female-gendered/sexed bladder cancer patients, leading to more personalized therapies.

#### Differences in metabolic enzymes

As previously discussed, mutations in bladder tumors can be caused by various environmental factors [107, 123]. Environmental carcinogens relevant to bladder cancer include nicotine-derived nitrosamine ketone (NNK), polyaromatic hydrocarbons (PAHs) from cigarette smoke, and arsenic. Multiple CYP450 enzymes and GSH are responsible for the detoxification of such carcinogens [124]. Several CYP450 enzymes are encoded by the *CYP1B1*, *GSTM1*, and *GSTP1* genes [125]. In bladder tumors, *CYP1B1*, *GSTM1*, and *GSTP1* are often mutated [126, 127]. Mutations in these genes deregulate toxin removal processes and therefore may potentially increase the risk of developing bladder cancer. Apart from the mutational deactivation of CYP450 enzymes, these enzymes can also be inhibited by sex hormones. Progesterone and estrogen inhibit the activity of CYP450 enzymes and subsequently contribute to the promotion of lung and breast cancer [128, 129]. In bladder cancer, the expression of progesterone is higher in female-gender patients compared with male counterparts [93]. The inhibition of *CYP450* enzymes by progesterone may dysregulate toxin removal in female-gender bladder cancer patients, promoting tumor progression.

### Differences in the male and female epigenome—both normal and tumor-bearing state

Epigenetic alterations of histones and DNA can lead to chromatin structure and gene expression changes [130]. The most well-studied epigenetic difference between men and women is DNA methylation in specific cell types within the human body. In healthy blood cells, there are sites in the genome that are differentially methylated between men and women [131–133]. In a recent study by Olivia et al., using whole blood cells to characterize the sex-based autosomal DNA methylation difference, they found female-sex have higher methylation in 76% of loci compared to male-sex [132]. The research indicates that there are underlying sex-specific differences in DNA methylation in both healthy as well as tumor-bearing states.

Altered DNA methylation at specific regions is a common mechanism in cancer. In bladder cancer, the overall genome is found to be hypomethylated, thereby providing increased genome stability and promoting tumor progression [134]. Hypomethylation is often linked with disease progression, whereas hypermethylation is associated with the silencing of the tumor suppressor genes. In bladder tumors, the promoter sites of genes associated with tumor-suppressive properties like apoptosis, cell invasion, DNA repair, and cell cycle control are found to be hypermethylated [135–137]. For example, hypermethylation in the promoter regions of *A3BP1*, *NPTX2*, *ZIC4*, *PAX5A*, *MGMT*, *IGSF4*, *GDF15*, *SOX11*, *HOXA9*, *MEIS1*, *VIM*, *STK11*, *MSH6*, *BRCA1*, *TBX2*, *TBX3*, *TERT*, *GATA2*, *DAPK1*, *CDH4*, *CCND2*, *GSTP1*, *CDKN2A*, *CDKN2B*, *WIF1*, and *RASSF1A* [135].

There are only two gender-associated altered DNA methylation studies conducted in bladder cancer until now. Charlotte et al., in 2010, identified that female-gendered bladder cancer patients had significantly higher rates of LINE1 hypomethylation compared to their male counterparts [138]. LINE1 hypomethylation is associated with increased bladder cancer risk [138]. There are a few mechanisms of LINE1 hypomethylation studied: [1] LINE1 hypomethylation increases the oxidative stress in bladder cancer, thereby promoting tumor progression [139] and [2] hypomethylation of the LINE1 promoter can induce an alternate splice variant of MET oncogene in bladder tumors, leading to tumor progression [140, 141]. Hypomethylation of LINE1 can thereby be one of the factors that lead to high rates of high-grade disease observed in female-gender bladder cancer patients. Another study conducted by Yuan et al. identified DNA methylation of the *DNA topoisomerase 2 beta* (*TOP2B)* gene mostly observed in male-gender bladder cancer patients compared with their female-gender counterparts [142]. The function of TOP2B in bladder cancer risk or progression currently remains undetermined. Valrubicin is a drug that interferes with the activity of topoisomerases such as TOP2B and is currently used as a treatment for BCG refractory NMIBC patients [142]. The potential side effect of valrubicin treatment is an increased risk of heart failure, but this risk can be suppressed by treatment with tamoxifen, an agonist of estrogen [143]. This can potentially suggest that the risk of side effects in male gender and female gender patients can differ based on the levels of estrogen in the tumor. Since the last 2 decades, only two gender-based DNA methylation studies have been conducted that show the disparity between male and female bladder tumor epigenome exists. There is a need to investigate the potential genes that are differentially hypo- or hypermethylated between male and female bladder tumors to understand whether they play a functional role in bladder cancer gender/sex disparities.

## Discussion

Sex and gender disparities exist in bladder cancer, where men have a four times higher incidence rate, and women have significantly higher rates of high-grade disease and a lower 5-year survival rate. Current research indicates that differences in immunological, hormonal, genetic, and epigenetic factors can contribute to these disparities, but the complete mechanism that causes these disparities is currently unknown. The in-depth analysis in this review suggests that there is a crosstalk between these biological factors, which can potentially explain some of the causes of the observed gender disparity. Future research to determine the elusive mechanism between these factors can help us elucidate promising targeted therapies in clinical settings, and validation work on such mechanisms will be necessary to advance the field and develop more effective, personalized approaches to bladder cancer treatment.

One way to validate the potential mechanisms that underlie sex disparities in bladder cancer is by utilizing in vivo models. To study tumor biology in vivo, animal models are developed by inducing the animals to certain conditions for the development of similar diseases to humans and mimicking the human anatomy, physiology, and TME [144]. The N-butyl-N-(4-hydroxy butyl)-nitrosamine (BBN) model is a commonly used carcinogen-induced animal model for studying bladder cancer. This model recapitulates the histology of human MIBC, overexpresses the markers of the basal cancer subtype, and has mutations in *Trp53*, *Kmt2d*, and *Kmt2c* that are common to those found in the human MIBC [80]. The BBN model holds significance in investigating the impact of sex hormones and immune responses in male and female bladder cancer patients, as it recapitulates human tumor biology and is immunocompetent [145].

Clinical, in vivo, and in vitro studies have demonstrated that age, race, immune responses, hormones, hormone receptor expression, the epigenetic and genetic alterations all can vary between tumors from male and female bladder cancer patients (summarized in Table 1). Here, we propose that certain factors such as hormone levels, immune cell composition, and immune response differ between men and women, and these differences distinctly predispose men and women to bladder cancer. After the development of a bladder tumor, these factors can also differentially influence tumor characteristics, the TME, and ultimately patient prognosis.

**Table 1:** Synopsis of gender/sex-related biasing factors: age, ethnicity, immune responses, hormonal influence, hormone receptor expression, epigenetic and genetic modifications variability in bladder cancer tumors across male and female patients.

Concept	Feature	Bias	Reference
Age	Metastasis in the lungs and liver	Older women	[16]
	Metastasis in bone and brain	Older men	[16]
Race	High rates of advanced-stage, high-grade, or metastatic disease	AA women	[25, 28, 29]
Tumor characteristics	Disease recurrence and progression	Women	[47, 48]
	High grade and stage at diagnosis	Women	[25, 26]
Immune system	Antibody immune response	Women	[55]
	CD4^+^ and CD8^+^ tissue-resident T cells	Women	[62]
	Chemokines (*Cx3xl1*, *Ccl2*, *Cxcl12*, and *Ccl5*)	Women	[62]
Hormone and hormone receptor expression	Progesterone receptor associated with high risk of cancer-specific mortality	Women	[93, 94]
Mutation	*PPARG* associated with aggressive bladder cancer	Women	[112–114]
	*TP53* is associated with cancer initiation, increased metastasis, disease progression, and proliferation	Men	[115–117]
	*KDM6A* associated with tumor progression	Women	[103, 105, 119–122]
Epigenome	Methylation of *TOP2B* gene	Men	[142]

Future studies should explore gender-specific therapies based on differentially mutated and differentially expressed genes. For example, female-gendered patients express higher levels of PPARG receptors compared with male-gendered patients and, therefore, may benefit more from treatment with PPARG modulators. As we know that there is a difference in the methylation pattern in the male and female epigenome, studies should utilize genomic technologies to identify differentially methylated CpG sites between male and female bladder cancer patients. This will guide the investigation of functional consequences of differential methylation, such as changes in gene expression that may influence bladder tumorigenesis or response to treatment. The immune response between healthy as well as tumor-bearing biological males and females varies, thus, studies should investigate whether we can capitalize on differences in the male and female immune systems when treating with immunotherapies. In this way, by understanding the mechanisms that drive bladder cancer gender/sex disparity, new strategies for personalized medicine can be developed. Finally, future studies should be conducted to identify whether gender identities outside of male and female experience disparities in bladder cancer. An improved understanding of the biological factors underlying gender disparities in bladder cancer has the potential to reveal new sex/gender-specific therapeutic vulnerabilities, which could ultimately benefit all patients.
